# Extracellular Vesicles From the Human Natural Killer Cell Line NK3.3 Have Broad and Potent Anti-Tumor Activity

**DOI:** 10.3389/fcell.2021.698639

**Published:** 2021-07-23

**Authors:** Allyson M. Cochran, Jacki Kornbluth

**Affiliations:** ^1^Department of Pathology, Saint Louis University School of Medicine, St. Louis, MO, United States; ^2^St. Louis VA Medical Center, St. Louis, MO, United States

**Keywords:** natural killer, extracellular vesicle, exosome, leukemia, NK3.3, NKLAM, breast cancer, ubiquitin ligase

## Abstract

Natural killer (NK) cells are critical mediators of immune function, responsible for rapid destruction of tumor cells. They kill primarily through the release of granules containing potent cytolytic molecules. NK cells also release these molecules within membrane-bound exosomes and microvesicles – collectively known as extracellular vesicles (EV). Here we report the characterization and anti-tumor function of EVs isolated from NK3.3 cells, a well described clonal normal human NK cell line. We show that NK3.3 EVs contain the cytolytic molecules perforin, granzymes A and B, and granulysin, and an array of common EV proteins. We previously reported that the E3 ubiquitin ligase, natural killer lytic-associated molecule (NKLAM), is localized to NK granules and is essential for maximal NK killing; here we show it is present in the membrane of NK3.3 EVs. NK3.3-derived EVs also carry multiple RNA species, including miRNAs associated with anti-tumor activity. We demonstrate that NK3.3 EVs inhibit proliferation and induce caspase-mediated apoptosis and cell death of an array of both hematopoietic and non-hematopoietic tumor cell lines. This effect is tumor cell specific; normal cells are unaffected by EV treatment. By virtue of their derivation from a healthy donor and ability to be expanded to large numbers, NK3.3 EVs have the potential to be an effective, safe, and universal immunotherapeutic agent.

## Introduction

Natural killer (NK) cells are a subset of lymphocytes with the innate ability to selectively recognize and kill cancer cells. They have both activating and inhibitory receptors that control their cytotoxic function. Tumor cells, by their down-regulation of self-recognizing major histocompatibility complex class 1 (MHC-I) molecules and expression of stress-induced proteins, shift the balance toward activation of NK cells. This induces migration and fusion of NK granules to the plasma membrane, followed by degranulation and entry of cytolytic molecules into the tumor cell, resulting in tumor cell death (Moretta and Moretta, 2004).

Granules arise from the formation of a dense core of effector molecules inside large, lipid membrane-bound vesicles within NK cells. Cytolytic proteins - perforin, granzyme A, granzyme B, and granulysin induce death in target cells by activating caspase -dependent and -independent death pathways (Sanchez-Ruiz et al., 2011). Upon fusion of granules with the plasma membrane, their contents are emptied into the immune synapse formed between the NK cell and the target cell. As a result, a concentrated discharge of potent effector molecules attack the target (Culley et al., 2009).

Within granules, smaller membrane-bound vesicles called exosomes are formed, which are secreted during degranulation. Activated NK cells also release microvesicles - pinched off sections of plasma membrane. All are collectively referred to as extracellular vesicles (EVs). Virtually all cells secrete EVs which function as a means for intercellular communication and modulation of cellular function via the diverse bioactive cargo within them. In addition to components common to all EVs, they also carry proteins, lipids, and RNA, unique to the cell type from which they originate. Exosomes are the smallest vesicles secreted by cells, ranging in size from 30 to 150 nm; microvesicles are larger, measuring between 100 and 1000 nm (Cufaro et al., 2019).

Extracellular vesicles derived from NK cells have been shown to contain nucleic acids (mRNA, micro (mi)RNA, rRNA) and common EV proteins such as CD63, tsg101, Alix, and HSP70. Importantly, NK-derived EVs also contain the cytolytic molecules responsible for NK-mediated anti-tumor activity. Recently, NK-derived EVs have been shown to effectively induce tumor cell death independent of the cell-to-cell contact necessary to activate the anti-tumor mechanisms of NK cells (Lugini et al., 2012; Jong et al., 2017; Zhu et al., 2017, 2019; Neviani et al., 2019; Wu et al., 2019; Federici et al., 2020).

We report the characterization and function of EVs derived from NK3.3. This cloned NK cell line was generated from the peripheral blood of a healthy human donor by our laboratory and is the only published NK cell line of this kind to date (Kornbluth et al., 1982, 1985; Kornbluth and Hoover, 1988). We performed comprehensive proteomic and RNA analysis of NK3.3-derived EVs. They contain the granule-associated proteins necessary for tumor killing, including natural killer lytic-associated molecule (NKLAM/RNF19b), an E3 ubiquitin ligase. Our laboratory identified NKLAM and found that it has two transmembrane domains and resides in the membranes of NK cell granules. Its ligase activity is mediated by three cysteine rich clusters that form a RING - in between RING - RING (RBR) domain. The catalytic activity of the RBR domain enables NKLAM to ubiquitinate its substrates. Studies conducted in our laboratory with NKLAM-deficient mice indicate that NKLAM is required for maximal NK-mediated anti-tumor function and for suppression of tumor dissemination and metastasis *in vivo* (Portis et al., 2000; Fortier and Kornbluth, 2006; Hoover et al., 2012; Lawrence et al., 2020).

Recent published studies have demonstrated tumor killing using exosomes derived from NK-92, an NK tumor line derived from a patient with non-Hodgkin’s lymphoma (Zhu et al., 2017). Other investigators used NK cells sorted and expanded from the peripheral blood of healthy human donors to collect NK-derived exosomes (Lugini et al., 2012; Jong et al., 2017). NK3.3-derived EVs reduce the need for identifying and consenting heathy peripheral blood donors as well as eliminating donor variability. They also prevent the potential introduction of tumorigenic elements that might occur using EVs from transformed NK-92 cells. Because use of NK-derived EVs is relatively new, there are a limited number of tumor types that have been examined for susceptibility to EV-mediated killing. These include melanoma (Zhu et al., 2017), neuroblastoma (Neviani et al., 2019; Wu et al., 2019), acute lymphoblastic leukemia (Wu et al., 2019; Di Pace et al., 2020) and glioblastoma (Zhu et al., 2018, 2019).

This study describes the anti-tumor effect of NK3.3-derived EVs. We initiated studies using K562 tumor cells, derived from the bone marrow of a patient with chronic myelogenous leukemia in blast phase, with characteristics of an acute leukemia (Lozzio et al., 1981). K562 cells are highly susceptible to NK-mediated killing because they lack expression of MHC-I (Kornbluth et al., 1985). We show that NK3.3-derived EVs induce morphological changes and altered protein expression in K562 characteristic of death via apoptosis. They inhibit tumor proliferation, and are strongly cytotoxic to K562 cells, while having no effect on the growth or survival of non-tumorigenic, normal cells. We performed proteomic analysis of K562 cells to identify changes in protein expression induced by NK EV treatment. We expanded studies to evaluate the ability of NK3.3-derived EVs to kill other types of tumor cells. We found that NK EVs kill T cell leukemia and breast cancer cells. This indicates that they have the potential to be a safe alternative, or synergistic partner, to current therapeutic modalities for multiple types of cancer, especially for those that lack effective, curative treatments.

## Materials and Methods

### Cell Lines

The human NK cell line, NK3.3, was maintained in RPMI-1640 medium supplemented with 10% FBS, 1% glutamine, 1% penicillin-streptomycin, and 200 IU/ml recombinant IL-2 (rIL-2) (R&D Systems). NK-92 cells (obtained from Dr. Hans Klingemann) were cultured using the NK3.3 culture protocol. Cryopreserved umbilical cord blood (CB) was obtained from the St. Louis Cord Blood Bank. Units were thawed and CD34+ hematopoietic stem cells were isolated using immunomagnetic CD34 antibody coated beads (Miltenyi Biotec). CD34+ cells were expanded in culture using a cocktail of cytokines (G-CSF, GM-CSF, IL-6, SCF, IL-7 and TPO) and aliquots of cells frozen over 2 weeks of culture. Peripheral blood mononuclear cells were obtained from normal donors as described (Kozlowski et al., 1999) and cultured for 3–7 days using the NK3.3 culture protocol. To obtain EV-depleted FBS, FBS was ultracentrifuged at 118,000 × *g* (Type 45 Ti rotor, Beckman Coulter, CA) for 20 h, then filtered through a 0.22 μm filter. Human embryonic kidney cells (HEK293) for EV collection were cultured in DMEM media supplemented with 3% EV-depleted FBS. K562 and Jurkat T cell leukemia cells (ATCC, Manassas, VA) were cultured in RPMI-1640 media supplemented with 7.5% FBS and 1% L-glutamine. HEK293 and breast cancer cell lines MCF7 and MDA-MB-231 (ATCC, Manassas, VA) were cultured in DMEM media supplemented with 1% glutamine, 1% penicillin-streptomycin, and 10% FBS. All cells were incubated at 37°C in a humidified atmosphere with 5% CO_2_.

### Extracellular Vesicle Generation and Isolation

NK3.3 and NK-92 cells were cultured overnight with IL-2 and then treated with phorbol myristate acetate (PMA) and ionomycin for 5 h to enhance secretion of EVs into the media. HEK293 cells were grown to 90% confluency over 48 h. Media was harvested and centrifuged at 300 × *g* for 10 min to pellet cells. A commercial polyethylene glycol polymer for precipitation of EVs was added to the supernatants for a minimum of 12 h at 4°C (ExoQuick-TC, System Bioscience (SBI)) and were centrifuged at 3000 × *g* at 4°C for 10 min per a modified ExoQuick-TC protocol. EV-depleted supernatants were discarded, and EV pellets were resuspended in PBS. Protein concentrations were assessed via BCA protein assay kit (ThermoFisher Scientific).

NK3.3-derived vesicle fractions were isolated by differential ultracentrifugation (Federici et al., 2020). Briefly, NK supernatants were centrifuged at 300 × *g* for 10 min, followed by centrifugation at 2,000 × *g* for 20 min and then 10,000 × *g* for 30 min at 4°C. This pellet was considered to be the microvesicle (MV) fraction. Additional ultracentrifugation at 100,000 × *g* for 90 min at 4°C was performed to isolate the exosome fraction. All pellets were resuspended in PBS and protein quantitated by BCA.

### Transmission Electron Microscopy Imaging

UV treated 300 mesh copper formvar coated grids were loaded with EV solution for 1.5 min, washed with 5 water drops, stained with 1% uranyl formate, blotted, and dried. EVs were imaged using a JEOL JEM1400 Plus transmission electron microscope (TEM) in the Research Microscopy and Histology Core, Saint Louis University.

### Nanoparticle Tracking Analysis

Size distribution of EV particles was determined by nanoparticle tracking analysis (NTA) using the NanoSight NS300 analyzer. Measurements were performed in triplicate for each EV preparation using 1 μg/μl EV protein diluted 1000-fold in PBS to obtain between 20 and 100 particles per frame. Samples were introduced at ambient temperature using an automated syringe pump set to 40, with camera level set to 13, detection threshold set to 3. Acquisition time was 60s and at least 200 tracks were completed. Data were obtained and analyzed using NTA 3.3 analytical software (Malvern Instruments).

### Proteomic and RNA Analysis

EV-containing preparations from NK3.3 cells were submitted to SBI for proteomic and RNA analysis. EVs were isolated, resuspended in buffer, and then filtered through a purification column (ExoQuick-TC ULTRA, SBI). For proteomic analysis, a 10 μg sample was processed by SDS-PAGE, the migration line excised, and in-gel digestion performed using trypsin. Analysis was performed using nano LC-MS/MS with a Waters NanoAcquity HPLC system interfaced to a ThermoFisher Q Exactive. Peptide sequence data was searched using Mascot software and filtered using Scaffold software.

For RNA analysis of the EV sample, RNA was extracted and purified using the RNA Purification Column kit (SBI). Small RNA libraries were constructed, purified, and then quantified. An 8% TBE gel was used to size select for the 140 to 300 bp region from the pooled libraries. This library was quantified by qPCR, and then high output single-end sequencing was performed.

For proteomic analysis, K562 cells were treated for 24 h with PBS (20% of total volume in culture media), HEK293 EVs or NK3.3 EVs (100 μg/ml). Cells were pelleted, washed, and then cell pellets were flash frozen on dry ice. Frozen pellets were submitted to Creative Proteomics. Cell pellets were lysed using an ultrasonic lysis device. Supernatants were transferred into YM-10 Microcon devices (Millipore) and centrifuged at 12,000 × *g* at 4°C for 10 min. A series of ammonium bicarbonate washes, reductions, and centrifugations were performed, the lyophilized extracted peptides were resuspended in formic acid, and 1 μg of protein was analyzed by LC-MS/MS using the Ultimate 3000 nano UHPLC system (ThermoFisher Scientific). The MS raw files were analyzed and searched against the human protein database based on sample species using Maxquant (1.6.2.6). Significance was defined as fold-change above 1.5 or below 1/1.5 determined by quantitative protein ratios.

### Pronase Assay

Twenty micrograms of NK3.3-derived EVs were treated with either PBS, Pronase (Sigma) at a final concentration of 10 μg/ml, or 10 μg/ml Pronase plus saponin (Sigma) at a final concentration of 0.2%, with final equivalent total volumes for all three conditions. Samples were incubated at 37°C for 1 h, mixed with Laemmli buffer, boiled at 95°C for 5 min, then immediately analyzed by immunoblotting.

### Cell Growth/Viability Assays

Cells were placed in fresh culture media 24 h before treatment. At the time of set up, cells were resuspended in fresh culture media at a final concentration of 5 × 10^5^ cells/ml – K562 cells, 2.5 × 10^5^ cells/ml – Jurkat cells, or 1 × 10^5^ cells/ml – HEK293, MDA-MB-231, and MCF7 cells. Treatments consisted of PBS only, EVs suspended in PBS, or staurosporine/PBS solution; all treatment volumes were 20% of the total culture volume. NK3.3-, or HEK293- derived EV treatment concentrations were between 0 and 100 μg/ml; the staurosporine concentration was 2.5 μM. Cells were incubated at 37°C in a humidified atmosphere with 5% CO_2_. Cells were evaluated morphologically and counted via hemocytometer using a phase microscope or automated cell counter (Countess II FL, Invitrogen) at 24, 48, and 72 h after treatment. Cell viability was assessed by trypan blue dye exclusion.

The WST-1 cell proliferation colorimetric assay (Roche) was used to quantitate the number of viable, metabolically active K562, Jurkat and IL-2 stimulated peripheral blood lymphocytes upon treatment with HEK293 or NK3.3 EVs. Cells were seeded in 96-well plates at 10,000 cells/well in a final total volume of 0.1 ml. Wells were set up in triplicate for each condition. At the desired time point, 10 μl of WST-1 was added to each well. After a 1 h incubation, absorbance was measured using a Gen5 microplate reader (BioTek) with a 450 nm filter.

Crystal violet (Sigma) was used to determine the ability of EVs to affect the viability and growth of adherent cell lines HEK293, MCF-7 and MDA-MB-231. Cells were seeded in 96-well plates at 15,000/well. At the desired time point, media was removed from the wells, wells were washed with PBS, and then incubated with 35 μl of crystal violet stain (0.1%) at room temperature for 10 min. After removal of the stain, 50 μl of lysing solution was added (0.1 M sodium citrate in 50% ethanol, pH 4.2) and incubated for 15 min. Absorbance was measured using a Gen5 microplate reader with a 590 nm filter.

K562 cells were cultured as previously described; CB cells were thawed and allowed to expand for 10 days before use. Metabolic activity was monitored using the RealTime-Glo MT Cell Viability Assay (Promega), with the kit protocol adapted for a 384-well plate with a final volume of 80 μl per well. Wells were seeded with 1,500 K562 or 5,000 CB cells. Final NK3.3 EV concentrations ranged from 25 to 50 μg/ml. Plates were placed in a Gen5 microplate reader and maintained at 37°C; luminescence output was measured at 1 h intervals for 89 h.

### Caspase Assay

Caspase-3 and -7 activity was measured using Caspase-Glo 3/7 (Promega). K562, Jurkat, MCF7 and MDA-MB-231 cells were treated with either 2.5 μM staurosporine, or 100 μg/ml of HEK293 EVs or NK3.3 EVs suspended in PBS at 20% total volume in Eppendorf tubes. 5,000 treated cells in 10 μl were added in triplicate to wells of 384-well plates for each condition and time point. At the appropriate time interval, 10 μl Caspase-Glo 3/7 substrate was added to each well. The plate was incubated for 1 h. For long term detection of caspase activity, 2.5 μM staurosporine or 100 μg/ml of HEK293 EV- or NK3.3 EV-treated K562 cells were seeded in 96-well plates at 50,000 cells/well triplicate for each condition and time point. Caspase activity, quantitated using Caspase-Glo 3/7, was determined using the kit protocol adapted for 5000 cells in 30 μl total volume for each reaction in 384-well plates. Luminescence was measured using the Gen5 microplate reader.

### Flow Cytometry

Cells were monitored for changes in cell surface CD71 expression using FITC anti-CD71 (M-A712, BD Biosciences). Apoptosis/cell death was determined using PE-Annexin V (#640908, BioLegend) with 7AAD staining (#559925, BD Biosciences). All assays were performed following manufacturers’ protocols and cells were analyzed using an LSRII flow cytometer (BD Biosciences). Data were evaluated using FlowJo software.

### Immunoblot Analysis

PBS- and EV-treated K562 cells were lysed in RIPA buffer (Pierce) supplemented with a protease inhibitor cocktail (ThermoFisher Scientific). Cell and EV lysates were analyzed by SDS-PAGE and transferred onto PVDF (Bio-Rad). Membranes were blocked using 3% BSA and probed with specific antibodies. Proteins were detected using either Clarity (Bio-Rad) or WesternBright (Advansta) western ECL blotting substrates via chemiluminescence (ChemiDoc XRS+, Bio-Rad). Protein loading was normalized using anti-β-actin antibody (A5441, Sigma). Antibodies used for this study include: Alix (#2171), annexin V (#8555), Bcl-2 (#2872), CD9 (#13174), cleaved caspases -3 (#9664), -7 (#8438), -8 (#9496), and -9 (#9505), granzyme A (#4928), granzyme B (#4275), HSP70 (#4876), HSP90β (#5087), ICAM1 (#4915), LAMP-1 (#3243), phospho-STAT1: Ser727 (#9177) and Tyr701 (#9167), STAT1 (#9172), and VCAM1 (#13662) - (Cell Signaling); cytochrome c (sc-13156), full-length caspases -3 (sc-7272) and -7 (sc-28295), granulysin (sc-271119), MHC-1 (sc-55582), perforin-1 (sc-136994), Tsg101 (sc-136111), DNAM-1 (sc-376736) - (Santa Cruz); CD63 (SBI); rabbit polyclonal anti-LAMP-1 tail antibody; mouse monoclonal NKLAM and MHC-II antibodies made in-house. Horseradish peroxidase-labeled secondary anti-mouse and anti-rabbit antibodies were from Cell Signaling.

### *In vivo* Tumor Studies

GFP-expressing MDA-MB-231 cells (2 × 10^6^) were injected into the 4th mammary fat pad of female athymic nude mice (Charles River). When tumors were palpable, 50 μg of either NK3.3 or HEK293-derived EVs were administered by intratumoral injection every 3–4 days. After 7 injections, mice were euthanized, tumors excised, formalin fixed, and paraffin embedded. Tumor sections were stained for apoptotic cells by terminal deoxynucleotidyl transferase dUTP nick end labeling (TUNEL), which detects DNA fragmentation. Slides containing tumor sections were scanned and fluorescent pixel intensity quantitated using CellSelect software (Takara Bio). Results are expressed as the ratio of TUNEL positive cells to total nuclei (stained with DAPI). Four control and 5 NK EV-treated mice were evaluated, with 3 tumor sections per mouse.

### Statistical Analysis

All data were recorded and analyzed in Microsoft Excel. Reported statistics are expressed as the mean ± SE. Statistical significance was determined at *p* < 0.05 using Student’s *t-*test with unequal variance.

## Results

### Verification of Extracellular Vesicle Isolation

**FIGURE 1:**
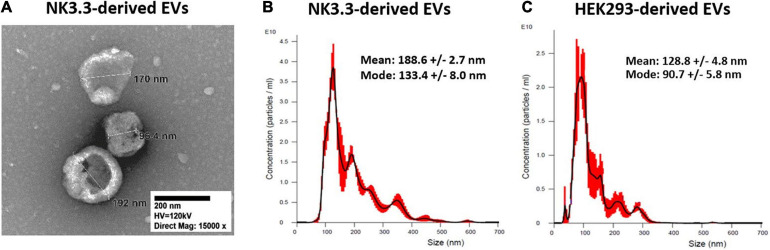
Size characterization of EVs derived from NK3.3 and HEK293 cells. **(A)** TEM was used to visualize and measure the size of EVs recovered from NK3.3 cells. EVs collected from NK3.3 **(B)** and HEK293 **(C)** supernatants were evaluated by NTA to measure vesicle size and particle concentration. Data are representative of 3 measurements each.

Transmission electron microscopy (TEM) and nanoparticle tracking analysis (NTA) were used to characterize the size and homogeneity of the vesicles isolated from NK3.3, NK-92, and HEK293 cells. Examination of TEM images of NK3.3-derived vesicles identified a mixed population of membrane-bound spherical structures, which coincide with the defined size ranges of both exosomes and microvesicles (Figure 1A). This mixed population will collectively be referred to as extracellular vesicles (EVs).

NTA concurrently identified a mixed population of vesicles produced by NK3.3 cells (Figure 1B). While the majority – represented by the highest peak – were 133 nm, consistent with the size of exosomes, the next highest peak signified a population of vesicles of 193 nm in size. For comparison, NTA was performed to evaluate vesicles produced by NK-92 cells (Supplementary Figure 1A). There was a less distinct division of size in this sample; the majority of NK-92 vesicles were 155 nm, closely followed by a population of 173 nm. Our measurements of NK-92 EVs corresponded to other published measurements for EVs derived from both NK-92 cells and NK cells isolated from peripheral blood mononuclear cells (Jong et al., 2017; Zhu et al., 2018, 2019; Federici et al., 2020). Vesicles produced by HEK293 cells were more homogeneous, with the majority measuring 91 nm, indicating that HEK293 EVs were primarily exosomes (Figure 1C). These measurements were consistent with other published HEK293 exosome measurements (Li et al., 2016). Therefore, NK3.3 EVs were similar in size to other NK-derived EVs. We obtained a high yield of NK3.3 EVs: 1.45 ± 0.13 × 10^12^ particles per 10^6^ cells, and 8.47 ± 0.98 × 10^10^ particles per μg (*n* = 23). This corresponds to 18.36 ± 1.00 μg per 10^6^ cells. A similar number of HEK293 EVs were obtained: 1.08 ± 0.09 × 10^11^ particles per μg (*n* = 23).

Differential ultracentrifugation was performed, following a standard protocol to separate microvesicles (MV) from exosomes (Federici et al., 2020). By NTA, the exosome fraction was almost identical to that obtained by polyethylene glycol polymer precipitation (Supplementary Figures 1B,C). The MV fraction contained larger particles and was more heterogeneous (Supplementary Figure 1D).

### Characterization of NK3.3-Derived Extracellular Vesicles

**FIGURE 2:**
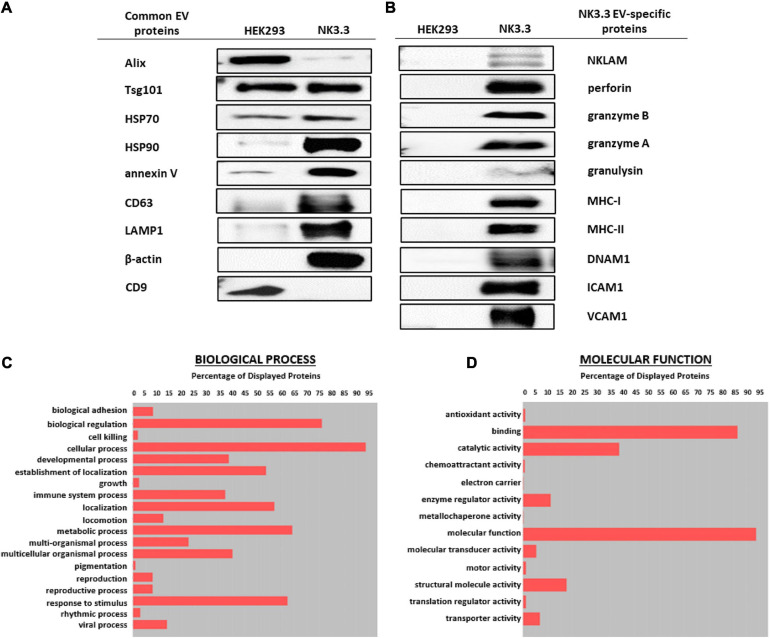
Comparison of EV proteins derived from NK3.3 and HEK293 cells. EV lysates were examined by immunoblot analysis using 30 μg of EV protein for **(A)** common EV proteins and **(B)** proteins specific to NK3.3 EVs. Gene ontology annotation generated from proteomic analysis identified the frequency of **(C)** biological processes and **(D)** molecular function associated with the proteins in NK3.3 EVs.

Immunoblot characterization of NK3.3- and HEK293-derived EVs indicates that they contained an array of EV-specific proteins, including CD63 and LAMP-1, heat shock proteins HSP70 and HSP90, vesicle structural proteins Alix and Tsg101 and other common EV cargo proteins β-actin and annexin V (Figure 2A). Published proteomic analysis of HEK293 EVs corroborates the expression of CD9 and other EV proteins (Li et al., 2016). Cytochrome-c, which resides between the inner and outer mitochondrial membrane, was detected in whole cell lysates; however, it was not found in EVs, making it one indicator of EV purity (Supplementary Figure 2A).

NK3.3-derived EVs also contained cytotoxic proteins required for NK anti-tumor activity: NKLAM, perforin, granzymes A and B, and granulysin, as well as immune-associated proteins MHC I and II and DNAX Accessory Molecule (DNAM-1; CD226), an activating receptor on NK cells (Neviani et al., 2019). NK3.3 EVs also expressed adhesion molecules ICAM1 and VCAM1 (Figure 2B). HEK293 EVs lacked the effector molecules possessed by NK3.3 EVs that contribute to cytotoxicity or participate in immune functions. For comparison, we assessed the expression of immune-associated proteins in NK3.3- and HEK293- EVs with NK-92 EVs, and in HEK293- and NK3.3-whole cell lysates (Supplementary Figure 2B). NK3.3- and NK-92- EVs expressed a similar array of proteins.

Proteomic analysis of NK3.3-derived EVs identified 623 proteins. Their subcellular distribution is shown in Supplementary Figure 2C. NK-associated proteins included granzymes A and B, perforin, MHC class I and class II molecules. Other proteins identified by proteomic analysis that were detected by immunoblotting included β-actin, Alix, CD63, various species of HSP70 and HSP90, ICAM1 and VCAM1. CCL5 (C-C motif chemokine 5/RANTES) was identified as a highly abundant protein and the most abundant chemokine. Another protein of interest was FCER1G (high affinity immunoglobulin epsilon receptor subunit gamma). This subunit is involved in signaling through multiple cell surface receptors. Also identified in the proteomic analysis was an array of surface proteins important in NK signaling and/or adhesion that may contribute to NK3.3 EV uptake and function: LFA-1 (CD11b/CD18), ezrin, CD70, CD59, CD53, CD50, CD48, CD47, and CD2 (Robertson et al., 1990; Montaldo et al., 2013). The distribution of proteins by biological process and molecular function is shown in Figures 2C,D. Proteins of interest in NK3.3 EVs associated with NK function are listed in Table 1. The complete proteomic dataset is included in the supplementary data (Supplementary Table 1). Based on the results of immunoblot and proteomic analysis, NK3.3 EVs contain a robust profile of death-inducing effector molecules and receptors that are used by NK cells to recognize, bind and kill tumor cells.

**TABLE 1:** Proteomic analysis of NK3.3-derived EVs listed by abundance.

Protein	Accession ID	Function
Actin	P60709	Cytoskeleton component
CCL5 (RANTES)	P13501	Chemokine
HLA class II DR, DQ	P01903, P01920	Antigen presentation
Granzyme B	P10144	Cytotoxic function
HSP90	P07900	Heat shock protein; chaperone
Granzyme A	P12544	Cytotoxic function
CD70	P32970	Cytokine; TNF family member; immune activation
Ezrin, moesin	P15311, P26038	Actin filament binding proteins
CD45	P08575	Hematopoietic cell membrane phosphatase
HLA class I A, B	P30512, P30495	Antigen presentation
FcRγ	P30273	CD16 signaling adapter protein
CD63	P08962	Tetraspanin EV protein
CD48	P09326	CD2 SLAM subfamily costimulatory molecule
CD59	P13987	Cell surface complement inhibition; CD2 ligand
Perforin	P14222	Cytotoxic function
HSP70	P0DMV8	Heat shock protein; chaperone
CD2	P06729	Costimulatory adhesion molecule
CD47	Q08722	Integrin-associated molecule; “don’t eat me”
ICAM1	P05362	Integrin binding transmembrane protein
CD53	P19397	Tetraspanin adhesion molecule
LFA-1 (CD11a/CD18)	P20701, P05107	Integrin; adhesion and costimulatory molecule
TNFSF4	P23510	Cytokine and chemokine regulation
CD82	P27701	Tetraspanin EV protein
CCR7	P32248	Chemokine
TNFRSF18	Q9Y5U5	Cytokine receptor; apoptosis; STAT1 phosphorylation
CD44	P16070	Cell adhesion; immune response
CD86	P42081	CD28 binding costimulatory molecule
CD166	Q13740	Leukocyte adhesion molecule
VCAM1	P19320	Integrin binding adhesion molecule
ICAM3	P32942	Integrin binding adhesion molecule
IL2Rβ	P14784	High affinity IL-2 receptor β chain
PDCD6IP (ALIX)	Q8WUM4	Exosome biogenesis

RNA profiling of NK3.3 EVs was also performed. Over 1000 mRNA species were identified in NK3.3 EVs. Among the most highly expressed were transcripts encoding membrane proteins and transport proteins. The next most represented RNA class was micro (mi)RNA; over 450 miRNAs were identified. Table 2 highlights some of the key miRNAs detected in NK3.3-derived EVs. The most abundant miRNAs were miR-21 and 21-5p, which are also the most abundant miRNAs in NK cells (Fehniger et al., 2010). Other highly expressed miRNAs were miR-155 and 155-5p, the miR-181 cluster, miR-92a-3p, hsa-let-7f, and miR146a-5p (Fehniger et al., 2010; Cichocki et al., 2011; Leong et al., 2012; Trotta et al., 2012; Pesce et al., 2018, 2020). Several of these miRNAs have tumor suppressor function. miR-16 has been identified as a tumor suppression factor in B cell leukemias (Aqeilan et al., 2010). miR-186 has been reported to inhibit neuroblastoma cells (Neviani et al., 2019). miR-146b-5p has been shown to inhibit drug-resistant colorectal cancer (Zhao et al., 2018). miR-34a suppresses acute myeloid leukemia (Wang et al., 2015). Another miRNA found in NK3.3 EVs, miR-3607-3p, inhibits pancreatic cancer cells (Sun et al., 2019). The frequency of the different RNA species is shown in Supplementary Figure 2D. The full RNA dataset of NK3.3 EVs is included in the supplementary data (Supplementary Table 2).

**TABLE 2:** List of abundant miRNAs in NK3.3-derived EVs.

miRNA	Chromosome	Function	References
miR-21-5p	17	Abundant in NK cells; regulates NK cell proliferation	Fehniger et al., 2010
miR-155-5p	21	Regulates IFNγ; tumor suppressor	Trotta et al., 2012
miR-181a-5p	1	NK cell development; promotes IFNγ	Cichocki et al., 2011; Leong et al., 2012
miR-181b-5p	9	NK cell development; promotes IFNγ	Cichocki et al., 2011; Leong et al., 2012
miR-92a-3p	13	NK cell maturation	Pesce et al., 2020
miR-146b-5p	10	Tumor suppressor	Zhao et al., 2018
miR-26b-5p	2	Growth inhibitor	Pesce et al., 2020
let-7f-5p	9	Cell development regulator; activates Toll-like receptors	Pesce et al., 2020
miR-146a-5p	5	Immune activator	Pesce et al., 2018
miR-16-5p	13	Abundant in NK cells; tumor suppressor	Aqeilan et al., 2010
miR-101-3p	1	Growth inhibitor	Pesce et al., 2020
miR-186-5p	1	Inhibits neuroblastoma growth	Neviani et al., 2019
miR-3607-3p	5	Tumor suppressor	Sun et al., 2019
miR-34a-5p	1	Immune activator; tumor suppressor	Wang et al., 2015

### Orientation of Membrane Proteins in NK3.3-Derived Extracellular Vesicles

**FIGURE 3:**
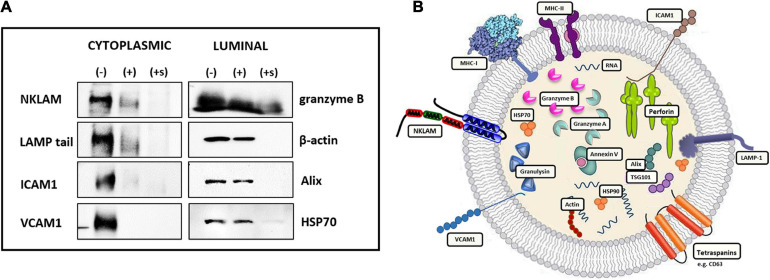
Orientation of transmembrane proteins in NK3.3-derived EVs. **(A)** Twenty micrograms of NK3.3 EVs were treated with either PBS (-), Pronase (+), or Pronase + saponin (+s). Samples were then boiled in Laemmli buffer; immunoblot analysis was performed using antibodies to protein epitopes that have a vesicle membrane, cytoplasmic-facing orientation or contained within the EV lumen. **(B)** Conceptualization of NK3.3 EV composition depicting the localization of proteins in NK3.3-derived EVs. The majority of NKLAM and LAMP-1 proteins are positioned in this orientation.

We utilized an enzymatic protein digestion assay to determine the orientation of several membrane proteins identified in NK3.3 EVs. NK3.3 EVs were treated with PBS (-), Pronase (+), or Pronase plus saponin (+s), then lysed and analyzed via immunoblotting (Figure 3A). Pronase, a combination of non-specific bacterial proteases, was used to digest proteins located on the surface - or cytoplasmic side - of the vesicle membrane. Addition of saponin permeabilized the vesicle membranes to allow Pronase to digest the proteins within the vesicle lumen.

NKLAM has two transmembrane domains. In NK lytic granules, both the N- and C-terminal ends of the protein are cytosolic-facing (Fortier and Kornbluth, 2006; Lawrence et al., 2020). We probed for NKLAM expression in EVs using an antibody that binds to an epitope on its C-terminal end (Kozlowski et al., 1999). Pronase treatment significantly diminished the expression of NKLAM; application of Pronase plus saponin completely eliminated antibody binding, indicating that the ubiquitin ligase domain of NKLAM is predominantly cytoplasmic-facing.

Other membrane proteins were evaluated using antibodies known to bind to extracellular or cytoplasmic-facing epitopes. The antibody used to probe for LAMP-1 binds to an epitope on its short C-terminal tail on the cytoplasmic side of membranes. Pronase treatment diminished LAMP1-tail expression. Application of Pronase plus saponin completely eliminated antibody binding, indicating that the majority of LAMP-1 is oriented with the C-terminus on the cytoplasmic face of the EVs. ICAM1 and VCAM1, both single-pass transmembrane adhesion proteins, are oriented on the cytosolic side of cellular membranes (Wai Wong et al., 2012). Both Pronase and Pronase plus saponin resulted in complete digestion of ICAM1 and VCAM1. This indicates that the portion of the proteins to which the antibodies bind is on the cytosolic/surface side of the EV membrane.

The combination of Pronase plus saponin was required for digestion of the luminal proteins examined: granzyme B, β-actin, Alix, and HSP70. Based on these results, we propose a model representing the location and content of components within NK3.3-derived EVs (Figure 3B).

### NK3.3-Derived Extracellular Vesicles Have a Potent Anti-Tumor Effect

**FIGURE 4:**
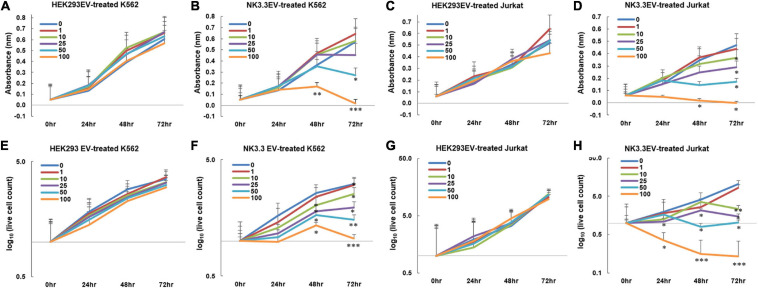
Inhibition of leukemia cell growth and metabolic activity by NK3.3-derived EV treatment *in vitro.* **(A–H)** Dose response analyses were performed using concentrations of EVs ranging from 1 to 100 μg/ml or PBS (0). Absorbance measurements using the WST-1 cell viability assay were assessed in K562 cells treated with either **(A)** HEK293 EVs or **(B)** NK3.3 EVs, and Jurkat cells treated with either **(C)** HEK293 EVs or **(D)** NK3.3 EVs. Mean absorbance ± SE; *n* = 3. Corresponding live cell counts, determined by automated cell counting using trypan blue dye exclusion, were performed at the same time as the WST-1 measurements for K562 cells treated with either **(E)** HEK293 EVs or **(F)** NK3.3 EVs, and Jurkat cells treated with either **(G)** HEK293 EVs or **(H)** NK3.3 EVs. Mean cell numbers ± SE; *n* = 3. *p-*Values determined by comparison of NK3.3 EV-treated cells at each treatment concentration to HEK293 EV-treated cells at the corresponding EV concentration. ^∗^*p* ≤ 0.05, ^∗∗^*p* ≤ 0.005, ^∗∗∗^*p* ≤ 0.0005.

Having established the presence of cytotoxic molecules and tumor suppressor miRNAs via immunoblot, proteomic and RNA analyses, we tested whether EVs derived from NK3.3 cells had an anti-tumor effect. K562 and Jurkat T cell leukemia cells were treated with increasing concentrations of either NK3.3- or HEK293- EVs. Low doses of NK3.3 EVs inhibited growth of K562 and Jurkat cells over 72 h, while high doses of NK3.3 EVs were cytotoxic. Equivalent doses of HEK293 EVs had no effect on K562 or Jurkat cells (Figures 4A–D). Therefore, the anti-tumor effect was dose dependent and specific to NK3.3-derived EVs.

We evaluated the effect of NK3.3 EVs on cell survival. The reduction in K562 live cell counts induced by NK3.3 EVs compared to HEK293 EVs was statistically significant beginning at 48 h with concentrations of 10 μg/ml and above. Treatment of Jurkat with concentrations of NK3.3 EVs at 25 μg and 100 μg/ml resulted in a significant reduction in live cells as early as 24 h (Figures 4E–H). Cells treated with 100 μg/ml NK3.3 EVs had the most rapid and continuous decrease in cell number over time.

NK3.3 EV treatment induced a variety of morphological changes in K562 cells (Supplementary Figure 3). Plasma membrane blebbing was observed in the majority of cells treated with NK3.3 EVs. This decreased with time and was replaced by the formation of apoptotic bodies within the cytoplasm, hypotonic swelling, and atypical structural changes and projections. We consistently observed these striking morphological changes that were both dose and time dependent.

We performed differential ultracentrifugation to assess the contribution of NK3.3-derived exosomes and microvesicles (MV) to the inhibition of K562. Using established protocols to separate exosomes from MV, we were not able to isolate the 110–120 nm vesicle population from the 180–220 nm population. However, the MV fraction contained a substantial number of particles in the 250–350 nm range (Supplementary Figures 1B–D). K562 cells were treated with each of these preparations; metabolic activity and live cell counts were monitored at 24 and 48 h. The NK3.3 exosome fraction had the strongest anti-tumor activity; the MV fraction was less effective (Supplementary Figures 4A,B).

**FIGURE 5:**
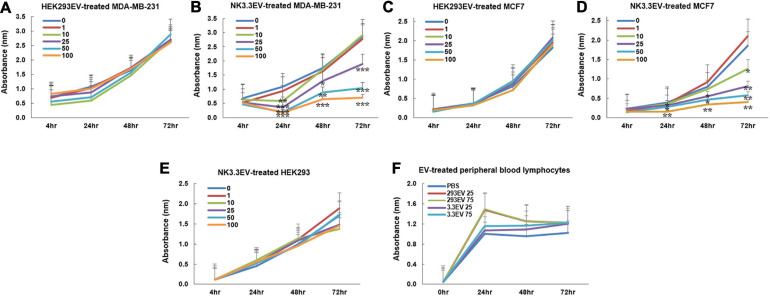
Inhibition of breast cancer cells but not non-tumorigenic cells by NK3.3-derived EV treatment *in vitro.* **(A–D)** Dose response analyses were performed using concentrations of EVs ranging from 1 to 100 μg/ml or PBS (0). Cells were monitored daily using a crystal violet viability assay. MDA-MB-231 cells were treated with either **(A)** HEK293 EVs or **(B)** NK3.3 EVs, and MCF7 cells treated with either **(C)** HEK293 EVs or **(D)** NK3.3 EVs. Mean absorbance ± SE; *n* = 3. **(E)** HEK293 embryonic kidney cells were treated with the same increasing NK3.3 EV concentrations and stained with crystal violet. **(F)** IL-2 stimulated peripheral blood lymphocytes from a healthy donor were treated with 25 or 75 μg/ml of either HEK293 EVs or NK3.3 EVs; metabolic activity was monitored using WST-1. Mean absorbance ± SE; *n* = 3. *p-*Values determined by comparison of NK3.3 EV-treated cells at each treatment concentration to HEK293 EV-treated cells at the corresponding EV concentration. *p ≤ 0.05, **p ≤ 0.005, ***p ≤ 0.0005.

Studies were expanded to evaluate the ability of NK3.3 EVs to inhibit breast cancer cells MDA-MB-231 and MCF7. MDA-MB-231 lack the expression of estrogen receptor (ER), progesterone receptor (PR) and human epidermal growth factor receptor 2 (HER2), classifying it as a triple-negative breast cancer cell line. MCF7 are ER-positive and PR-positive and are considered to be less aggressive and invasive than MDA-MB-231 cells (Cailleau et al., 1978). As shown in Figures 5A–D, NK3.3-derived EVs reduced the viability of both breast cancer cell lines in a dose and time-dependent manner; comparable amounts of HEK293 EVs had no effect.

### NK3.3-Derived Extracellular Vesicles Do Not Affect Survival of Non-Tumor Cells

NK3.3 EVs were examined for their potential toxicity against normal or non-tumorigenic cells. We treated the human embryonic kidney line HEK293 and IL-2 stimulated peripheral blood lymphocytes or cord blood lymphocytes with NK3.3-derived EVs. There was no significant change in metabolic activity or viability with NK EV treatment (Figures 5E,F and Supplementary Figure 4). This reflects the selective anti-tumor cytotoxic activity of NK3.3 EVs.

### NK3.3-Derived Extracellular Vesicles Induce Apoptotic Death of Tumor Cells

To identify the death pathways induced by NK3.3 EVs, we performed an annexin V apoptosis assay. PBS- and NK3.3 EV-treated K562 and Jurkat cells were stained with fluorochrome-conjugated annexin V (PE-Annexin V) and vital dye (7AAD) to identify early apoptosis and late apoptosis/necrosis, respectively. Treated cells were analyzed by flow cytometry over the course of treatment.

**FIGURE 6:**
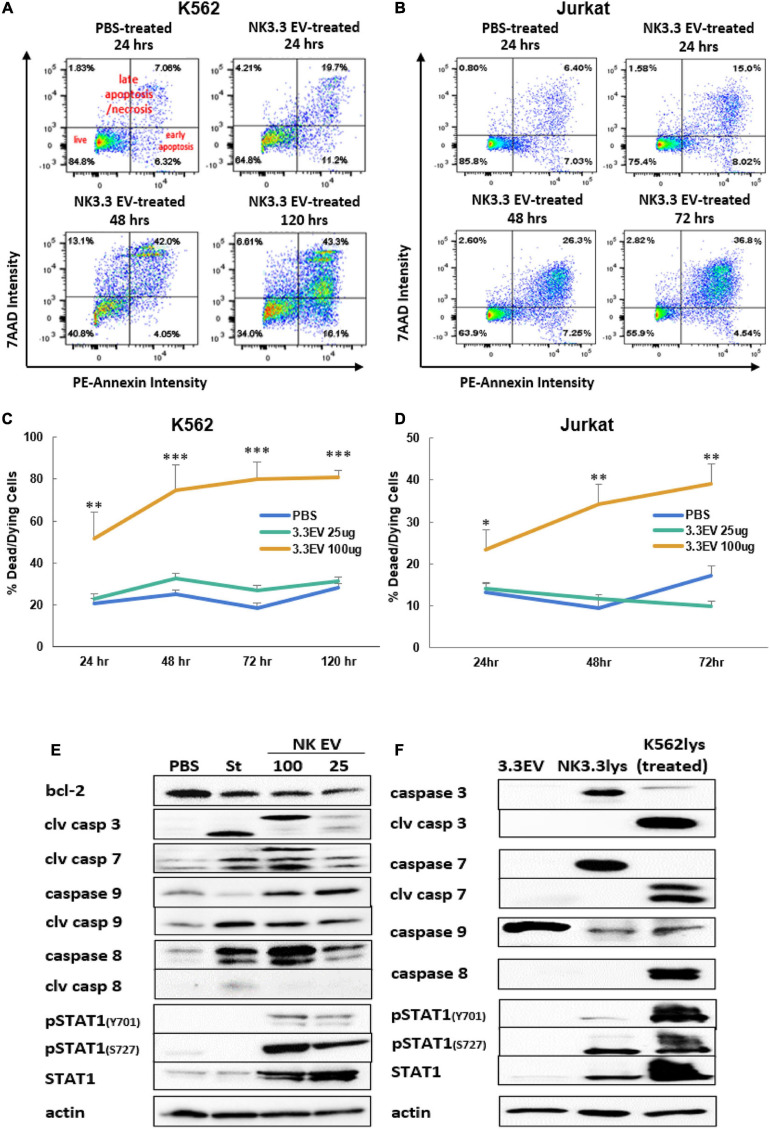
Induction of apoptosis in leukemia cells and alterations in K562 protein expression by treatment with NK3.3-derived EVs. Leukemia cells treated with either PBS, 25 or 100 μg/ml NK3.3 EVs were stained for annexin V and 7-AAD and analyzed by flow cytometry. Representative scatter plots are presented for **(A)** K562 cells and **(B)** Jurkat cells. Graphs of the cumulative apoptosis data collected by flow cytometry are shown for **(C)** K562 cells and **(D)** Jurkat cells. Dying/dead cell frequency is the sum of all annexin V- and 7AAD-positive quadrants. Mean frequency ± SE; *n* = 6. ^∗^*p* ≤ 0.05, ^∗∗^*p* ≤ 0.005, ^∗∗∗^*p* ≤ 0.0005. **(E)** Immunoblots of K562 protein lysates from cells treated for 24 h with PBS, 2.5 μM staurosporine (St), 100 μg/ml NK3.3 EVs (100), or 25 μg/ml NK3.3 EVs (25); 25 μg protein per lane was used. **(F)** Comparison of protein expression from NK3.3-derived EVs (3.3EV), NK3.3 cells (NK3.3lys), or K562 cells 24 h post- 100 μg/ml NK3.3 EV treatment (K562lys). Lysates were prepared, and immunoblotting performed to assess the potential contribution of NK EV-associated proteins to NK EV-treated K562 cells (25 μg of protein per lane).

Scatter plots from representative experiments show the frequency of live cells in the lower left quadrants, early apoptosis in the lower right quadrants, and late apoptosis/necrosis in the upper quadrants (Figures 6A,B). The upper left plot shows PBS-treated cells at 24 h. Greater than 80% of the PBS-treated control cells were alive with minimal, normally occurring death common for these cell lines. Over the time course of NK EV treatment, there was a progressive reduction in live cells, an increase in apoptotic cells, and a shift from apoptosis to cell death/necrosis.

The full data set of the cytotoxic effect of NK3.3 EVs on K562 and Jurkat is presented in Figures 6C,D. The most dramatic cell death occurred in cells treated with 100 μg/ml NK3.3 EVs at all time points, with statistical significance beginning as early as 24 h. By 72 h, over 80% of the remaining K562 cells and 40% of remaining Jurkat cells were dead or dying. The results of this analysis indicate that NK3.3-derived EVs induced death of K562 and Jurkat tumor cells in a time and dose dependent manner.

### NK3.3-Derived Extracellular Vesicles Affect Expression of Apoptosis-Associated Proteins

Immunoblotting was performed to identify changes in expression of key proteins involved in cell death pathways. K562 lysates were prepared 24 h after treatment with PBS, the apoptosis inducer staurosporine or NK3.3 EVs. Caspases -3, -7, -9, and their cleavage products - essential to intrinsic apoptosis pathway function - were evaluated. Treatment with NK3.3 EVs at 100 μg/ml or staurosporine induced the greatest expression of these three caspase cleavage products (Figure 6E). Both treatments increased cleaved caspase-3 by 20-fold, cleaved caspase-7 by 20-fold and 6-fold, respectively, and cleaved caspase-9 by 2.5-fold compared to PBS-treated cells. NK3.3 EV treatment at 25 μg/ml produced the same caspase cleavages, but to a lesser extent. Both NK EV treatments reduced the expression of Bcl-2, an important pro-survival protein associated with mitochondrial involvement in apoptosis (Ben Safta et al., 2015), by 50%.

We also investigated the expression of other proteins associated with apoptosis. Caspase-8 was upregulated in all treated conditions; 100 μg/ml NK3.3 EV treatment induced upregulation by more than 40-fold. However, only staurosporine treatment produced an active caspase-8 cleavage product. STAT1, a transcription factor shown to have an association with apoptosis via its interaction with caspases -3 and -7, was phosphorylated at tyrosine701 and serine727 when K562 cells were treated with NK3.3 EVs; no phosphorylation was induced by staurosporine treatment. Total STAT1 was also highly upregulated in cells treated with NK3.3 EVs, while staurosporine-treated cells expressed the same basal level of STAT1 as PBS-treated cells.

To determine the contribution of EV-derived proteins to the results observed, we examined the protein composition of NK3.3 EV lysates, NK3.3 whole cell lysates, and whole cell lysates of K562 cells treated with NK3.3 EVs for 24 h. NK3.3 EVs did not contain caspases -3, or -7, or their cleavage products (Figure 6F). NK3.3 EVs also did not contain caspase-8, cleaved caspase-8, or phosphorylated STAT1. There was, however, a small amount of total STAT1 in NK3.3 EVs. NK3.3 EVs did contain a relatively large amount of procaspase-9 compared to that found in NK3.3- and K562- cell lysates. Taken together, these results suggest that NK3.3-derived EVs induced death in K562 cells largely through an intrinsic apoptosis program than by extrinsic apoptosis.

### NK3.3-Derived Extracellular Vesicles Induce Significant Caspase 3/7 Activity

**FIGURE 7:**
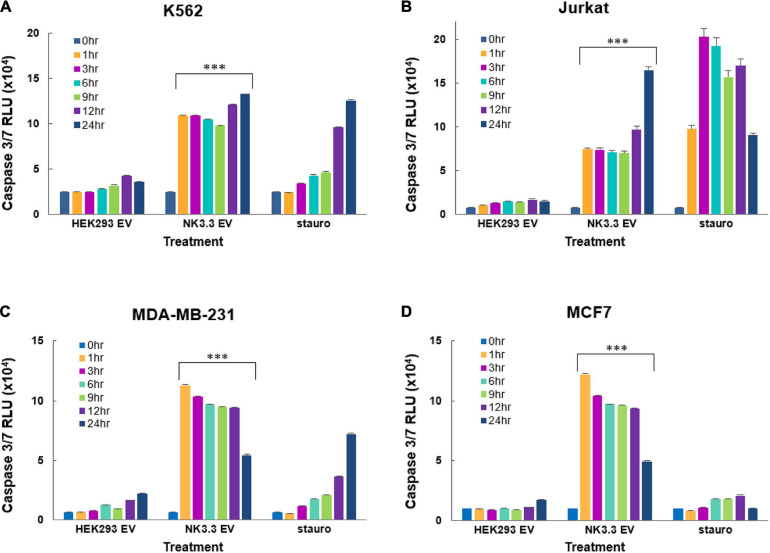
Induction of early caspase-3/7 activity in cancer cells treated with NK3.3-derived EVs. Leukemia cells and breast cancer cells were treated with either 100 μg/ml HEK293 or NK3.3 EVs or 2.5 μM staurosporine. A DEVD pro-luciferin substrate was added to **(A)** K562, **(B)** Jurkat, **(C)** MDA-MB-231, and **(D)** MCF7 cells at the indicated times. A luminescent signal is generated upon cleavage by activated caspases -3 and -7. RLU: relative luminescence unit. Mean luminescence ± SE; *n* = 6. *p-*Values determined by comparison of NK3.3 EV-treated cells to HEK293 EV-treated cells. ^∗∗∗^*p* ≤ 0.0005.

We assessed the induction of caspase -3 and -7 activity by NK3.3 EV treatment of K562, Jurkat, MDA-MB-231 and MCF7 cells using the luminescent Caspase-Glo 3/7 assay. NK3.3 EVs induced significant amounts of active caspases 3/7 in all tumor cell lines at all time points (1–24 h) when compared to both PBS- and HEK293 EV-treated cells (Figure 7). Caspase 3/7 activity remained high in K562 over 72 h (Supplementary Figure 4D).

### NK3.3-Derived Extracellular Vesicles Inhibit Proliferation of Tumor Cells

**FIGURE 8:**
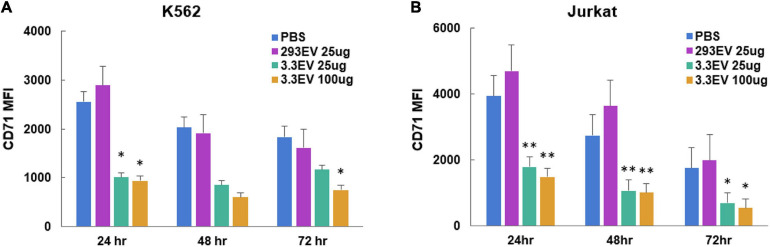
Reduction in expression of leukemia cell proliferation marker CD71 in NK3.3 EV treated cells. **(A)** K562 and **(B)** Jurkat cells were treated with either PBS, 25 μg/ml HEK293 EVs, 25 or 100 μg/ml NK3.3 EVs, stained for proliferation marker CD71, and analyzed by flow cytometry. MFI: mean fluorescence intensity. Mean MFI ± SE; *n* = 3. *p-*Values determined by comparison of NK3.3 EV-treated cells to HEK293 EV-treated cells. ^∗^*p* ≤ 0.05, ^∗∗^*p* ≤ 0.005.

To evaluate the effect of NK3.3 EVs on tumor proliferation, we measured the change in cell surface CD71 expression via flow cytometry. CD71 (transferrin receptor-1) is commonly used as a proliferation marker; its expression decreases when proliferation is inhibited (Caruso et al., 1997). Analysis of mean fluorescence intensity (MFI) indicated that there was a significant reduction in CD71 expression by K562 and Jurkat cells treated with either 25 or 100 μg/ml NK3.3 EVs as early as 24 h after exposure (Figure 8). Statistically significant reductions in CD71 expression were seen in both K562 and Jurkat cells treated with either concentration of NK3.3 EVs over 72 h.

### Proteomic Analysis of K562 Tumor Cells Treated With NK3.3-Derived EVs

Proteins from K562 cells treated with PBS, 100 μg/ml HEK293- or NK3.3-derived EVs for 24 h were profiled by mass spectrometry. 2769 proteins were identified; 492 proteins changed at least 1.5 fold with NK3.3 EV treatment. Table 3 is a list of selected, highly upregulated or downregulated proteins in NK3.3 EV-treated K562 cells when compared to HEK293 EV-treated cells. The full proteomic dataset is included in the supplement (Supplementary Table 3).

**TABLE 3:** Proteomic analyses of NK3.3 EV-treated K562 cells.

Protein name	Gene name	Change in protein expression
Granzyme B	*GZMB*	UP
Leukocyte surface antigen CD47	*CD47*	UP
Inhibitor of nuclear factor kappa-B kinase-interacting protein	*IKBIP*	UP
Integrin beta-3	*ITGB3*	UP
CD44 antigen	*CD44*	UP
Intercellular adhesion molecule 1	*ICAM1*	UP
Interferon-induced guanylate-binding protein 2	*GBP2*	UP
Signal transducer and activator of transcription 1-alpha/beta	*STAT1*	UP
N-myc-interactor	*NMI*	UP
Integrin alpha-5; heavy chain; light chain	*ITGA5*	UP
Transforming growth factor beta-1	*TGFB1*	UP
NF-kappa-B-repressing factor	*NKRF*	UP
Apoptosis regulator BAX	*BAX*	UP
Mini-chromosome maintenance complex-binding protein	*MCMBP*	DOWN
Sororin	*CDCA5*	DOWN
Geminin	*GMNN*	DOWN
Cyclin-dependent kinase 4	*CDK4*	DOWN
Protein tyrosine phosphatase type IVA 2; type IVA 1	*PTP4A2; PTP4A1*	DOWN
DNA replication complex GINS protein SLD5	*GINS4*	DOWN
Cell division cycle protein 23 homolog	*CDC23*	DOWN
PCNA-associated factor	*KIAA0101*	DOWN
BRCA1-associated ATM activator 1	*BRAT1*	DOWN
DNA polymerase alpha subunit B	*POLA2*	DOWN
Cell division control protein 45 homolog	*CDC45*	DOWN
Cell division cycle protein 123 homolog	*CDC123*	DOWN
Proliferating cell nuclear antigen	*PCNA*	DOWN
Transferrin receptor protein 1	*TFRC*	DOWN

Granzyme B was one of the most abundantly expressed proteins detected in NK3.3 EV-treated cells. Control-treated cells contained no detectable granzyme B, indicating that granzyme B was most likely introduced by NK EVs. CD47, which functions as a “don’t eat me” signal (Takimoto et al., 2019), was highly expressed in NK3.3 EV-treated K562 cells compared to control-treated cells. NK3.3 EVs also contain CD47, which could be, in part, responsible for the increased expression detected in K562 cells. The adhesion molecules CD44, ICAM1, integrin β-3 and integrin α-5, were also highly abundant (Wai Wong et al., 2012; Anderson et al., 2014). Inhibitor of nuclear factor kappa-B kinase-interacting protein, another highly upregulated protein, as well as apoptosis regulator BAX, are associated with promoting apoptosis and cell death (Pawlowski and Kraft, 2000; Hofer-Warbinek et al., 2004). Other upregulated proteins such as STAT1, N-myc-interactor, TGFβ-1, and NF-kappa-B-repressing factor, are associated with inhibition of proliferation and/or transcription (Niedick et al., 2004; Dimco et al., 2010; Kubiczkova et al., 2012; Pruitt et al., 2016).

The protein with the most reduced expression was mini-chromosome maintenance complex-binding protein (MCMBP), which is involved in maintaining sister chromatid cohesion and whose increased expression is linked to highly proliferative malignant cells (Quimbaya et al., 2014). A large majority of the other proteins with greatly decreased expression listed in Table 3: sororin, geminin, CDK4, PTP4A2/A1, GINS4, CDC23, PCNA-associated factor, BRAT1, POLA2, CDC45, CDC123, PCNA, and CD71, are closely associated with DNA replication mechanisms and proliferation (Caruso et al., 1997; Masai et al., 2010; Quimbaya et al., 2014). These results reinforce the finding that NK3.3-derived EVs induce cell death and inhibit tumor cell proliferation via multiple mechanisms.

### NK3.3 EVs Induce Apoptosis of Breast Cancer Cells *in vivo*

**FIGURE 9:**
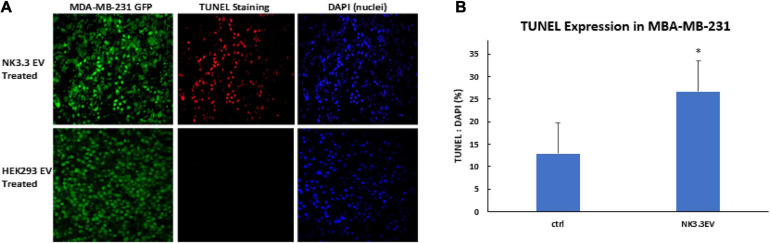
Apoptosis induction in MDA-MB-231 breast cancer cells by intratumoral injection of NK3.3 EVs *in vivo*. **(A)** GFP-expressing MDA-MB-231 tumors in the mammary fat pad of female nude mice were excised, paraffin embedded, sectioned, and subjected to TUNEL staining (red). Tumor cells: GFP+ (green); Nuclei: DAPI+ (blue). EVs from HEK293 human epithelial cells served as a negative control. **(B)** Comparison of TUNEL staining of tumors from control and NK3.3 EV-treated mice. Whole slide scanning was performed, and fluorescence intensity was quantitated using CellSelect software. Results of tumor sections from 4 control and 5 NK3.3 EV-treated mice are presented as % TUNEL/DAPI positive. ^∗^*p* < 0.05.

To determine whether NK3.3 EVs have cytotoxic activity *in vivo*, GFP-expressing MDA-MB-231 cells were injected into the mammary fat pad of female nude mice. When tumors were palpable, NK3.3 or HEK293-derived EVs were injected intratumorally every 3–4 days. After 7 injections, tumors were excised, formalin fixed and paraffin embedded. Tumor sections were analyzed for apoptotic cells by TUNEL staining, to detect DNA fragmentation, and DAPI, to label nuclei. Representative staining is shown in Figure 9A. Fluorescent pixel intensity was quantitated in tumors from 4 control-treated and 5 NK3.3 EV-treated mice (3 tumor sections per mouse). Results are expressed as the ratio of TUNEL positive cells to total nuclei. As shown in Figure 9B, there was significantly more apoptosis in breast tumors treated with NK3.3 EVs than in the control-injected mice.

## Discussion

We report here the results of a comprehensive analysis of the characteristics and function of extracellular vesicles derived from NK3.3 cells (Kornbluth et al., 1982). NK3.3 EVs induce robust anti-tumor activity *in vitro*. They activate both anti-proliferative and apoptotic mechanisms in an array of tumor cell lines while having no effect on normal cells. As a first step toward the development of NK3.3 EVs for cancer treatment, we demonstrate that NK3.3 EVs induce apoptosis of triple negative breast cancer cells *in vivo*. We identify key NK3.3 EV proteins by both immunoblotting and proteomic analysis that are cytolytic effector molecules. Next generation RNA sequencing analysis identified miRNA species that have tumor-suppressing activity. We also performed proteomic and immunoblot analysis of tumor cells treated with NK3.3 EVs, which identified multiple pathways associated with proliferation and cell death.

One component with a critical role in NK anti-tumor activity is NKLAM (Kozlowski et al., 1999; Portis et al., 2000; Fortier and Kornbluth, 2006; Hoover et al., 2012; Lawrence et al., 2020). We show for the first time that NKLAM is a component of NK EVs. Pronase digestion experiments indicate that the N- and C- terminal ends of NKLAM in the EV membrane are primarily positioned facing the cytoplasm. Orientation of NKLAM with its ubiquitin ligase domain facing the cytosol indicates that this is where interaction with substrates and ubiquitination occurs. Studies are currently ongoing to identify NKLAM targets of ubiquitination within tumor cells.

In this study, NK-derived EVs were generated from a non-tumorigenic NK cell line, NK3.3, isolated from the peripheral blood of a healthy donor. NK3.3 cells have been thoroughly characterized (Kornbluth et al., 1982, 1985; Kornbluth and Hoover, 1988; Kozlowski et al., 1999). While studies using NK-derived exosomes have emerged in the last few years, none use a cloned, normal human NK cell line. NK-derived EVs have been isolated and evaluated from B6 mice, human NK cells collected from peripheral blood, or tumorigenic non-Hodgkin’s lymphoma NK cells (NK-92) (Lugini et al., 2012; Jong et al., 2017; Zhu et al., 2017, 2019; Neviani et al., 2019; Wu et al., 2019; Federici et al., 2020). Although NK-92 -derived EVs have anti-tumor activity, studies have shown that EVs derived from transformed/tumorigenic cells carry cargo specific to those cells that are capable of negatively influencing or altering recipient cells (Hannafon and Ding, 2013; Mashouri et al., 2019). Therefore, NK3.3-derived EVs are likely to be safer and more effective for therapeutic use.

Comparison of NK3.3 EV size to NK-92- and peripheral blood NK cell- derived EVs show strong similarities. By NTA, we identified a major population of NK3.3 EVs in the range of 120–130 nm, with a smaller population in the 170–195 nm range. Our measurements correspond to NK-92 EV measurements taken in our laboratory as well as NK-92- and peripheral blood NK- EV published data (Jong et al., 2017; Zhu et al., 2017). In those reports, NK EVs ranged from 100 to 170 nm, consistent with the size of the vesicles we obtained from NK3.3. It is likely that our EV preparations contain both exosomes and MV. Ultracentrifugation at 10,000 × *g* to isolate MV, with further ultracentrifugation at 100,000 × *g* to obtain exosomes, was performed. MV ranged from 129 to 344 nm. The exosome fraction was identical to that obtained using polyethylene glycol polymer precipitation. Functional assays indicated that the strongest anti-tumor activity was mediated by the exosome fraction. The NK3.3 EV protein profile also closely compares to published NK-92- and peripheral blood NK- EV content. Common EV proteins CD63, tsg101, HSP70, and Alix, and primary effector molecules perforin, granzymes A and B, and granulysin have been identified in all three types of NK EVs (Lugini et al., 2012; Jong et al., 2017; Zhu et al., 2017; Neviani et al., 2019; Wu et al., 2019). We are the first to report full proteomic analysis of NK3.3 EVs, which identified NKLAM as well as several additional proteins that play a critical role in NK EV function.

NK3.3 EVs are cytotoxic to several different types of tumor cells while having minimal effect on normal cells. Protein profiling of NK3.3 EVs identified high levels of adhesion molecules ICAM1 and VCAM1, which may participate in EV binding and/or uptake. Recent studies have shown that DNAM-1 is expressed by NK exosomes. Blocking of DNAM-1 on the NK exosome delayed tumor apoptosis (Di Pace et al., 2020). Therefore, expression of DNAM-1 on NK3.3 EVs may play a role in their selective anti-tumor activity. Additional candidates within the NK3.3 EV proteome involved in NK cell adhesion and signaling were also identified. CD50 (ICAM3) and ICAM1 are ligands of LFA-1, which contributes to immune cell binding, migration, and signal activation (Montaldo et al., 2013). CD53 amplifies NK cell adhesion by enhancing the activation of LFA-1 (Todros-Dawda et al., 2014). CD48 and CD59 are ligands for CD2 (Deckert et al., 1992). The interaction between CD48 and CD2 has been shown to stimulate signaling for NK cell degranulation. CD2 also promotes the formation of plasma membrane nanotubes which NK cells use to tether to target cells and transmit molecular information (Comerci et al., 2012). Ezrin and CD47 contribute to adhesion and migration (Oldenborg, 2013; Takimoto et al., 2019). CD70 participates in NK cell adhesion through interaction with the CD27 receptor (Yang et al., 1996); both CD47 and CD70 also have a role in apoptosis signaling (De Colvenaer et al., 2010; Oldenborg, 2013). CD47 was one of the most abundantly expressed proteins in NK3.3 EV-treated K562 cells. Determination of the molecules involved in the interaction between EVs and tumor cells is under investigation.

Novel proteins identified in NK3.3 EVs include the chemokine CCL5/RANTES and high affinity immunoglobulin epsilon receptor subunit gamma FCER1G. CCL5 is involved in the recruitment of lymphocytes and monocytes to sites with pathogenic antigens or abnormal cells like tumors (Aldinucci and Colombatti, 2014). In an *in vivo* setting, areas of high concentrations of NK-derived EVs may amplify the immune response within the tumor microenvironment. The same amplification response may hold true for FCER1G in that it can associate with surface receptor complexes to initiate a killing response in recruited immune cells. Further studies of the role of proteins identified in this analysis in the anti-tumor activity of NK EVs are warranted.

NK3.3 EVs upregulate and phosphorylate STAT1 in K562 cells, which may be another mechanism for inhibiting proliferation and inducing killing of K562 cells. NK3.3 EV treatment stimulates phosphorylation of STAT1 at serine-727 and at tyrosine-701. IFN-γ is the primary ligand for STAT1 activation through the induction of tyr701 phosphorylation, which is associated with regulation of pro-apoptotic gene expression (Stephanou and Latchman, 2003). IFN-γ has been shown to be a component of NK EVs (Di Pace et al., 2020). STAT1 has also been shown to inhibit proliferation by reducing cyclin D expression (Dimco et al., 2010). High levels of caspases -3 and -7 activity were found in cells expressing a constitutively active form of STAT1 (Sironi and Ouchi, 2004). Therefore, NK3.3 EV activation of STAT1 in K562 cells might enhance apoptosis in both a caspase -dependent and -independent manner while also upregulating expression of anti-proliferation and/or pro-apoptosis genes. Identification of STAT1 gene targets in NK3.3 EV-treated K562 cells remains to be determined.

RNA sequencing analysis identified a diverse profile of RNA species. Some of the miRNAs identified in NK3.3 EVs are also found in NK cells, where they regulate NK function, granzyme B and perforin expression, and IFN-γ production (Fehniger et al., 2010; Trotta et al., 2012). This profile is markedly different from the miRNA profile reported for HEK293-derived EVs (Li et al., 2016). Other miRNAs identified in NK3.3 EVs are miR-16, miR-3607-3p and miR-186. miRNAs miR-16, miR-3607-3p and miR-186 have been shown to have tumor suppressor activity. One target of miR-16 is Bcl-2 (Yan et al., 2013); miR-186 from peripheral blood NK-derived exosomes has been shown to inhibit proliferation of neuroblastoma cells by targeting TGFβ1 and other proliferation genes (Neviani et al., 2019). miR-3607-3p was found to inhibit proliferation, migration, and invasion of pancreatic cancer cells, potentially by targeting IL-26 (Sun et al., 2019). Of the mRNAs identified, the transcript for HUWE1 was the eleventh most abundant. HUWE1 is a HECT E3 ubiquitin ligase that ubiquitinates and degrades the anti-apoptotic protein Mcl-1, suggesting it might play a role in the anti-tumor activity of NK3.3 EVs (Strappazzon et al., 2019). More extensive mining of the RNA data is underway to identify additional RNA transcripts in NK3.3 EVs that might cause tumor suppression.

NK3.3 EVs induce caspase 3/7 activity in all the tumor lines tested. NK3.3 EVs contain high concentrations of perforin and granzymes B and A. Once taken up by the target cell, granzymes enter the cytoplasm to induce apoptosis via disruption of mitochondrial outer membrane potential and cleavage of caspases -3, -7, and -9 (Metkar et al., 2002; Bots and Medema, 2006). Granzyme B directly targets and cleaves caspases -3 and -7, leading to a rapid initiation of apoptosis. It also induces activation of the intrinsic apoptosis pathway by cleaving BID to tBID, initiating a disruption of the mitochondrial outer membrane potential and release of cytochrome c. This is followed by formation of apoptosome scaffolding that incorporates caspase-9, which is then cleaved into active caspase-9 within the apoptosome. This active structure then moves on to cleave caspases -3 and -7 into active products. Many of these cleavage products translocate to the nucleus where they induce DNA damage (Ben Safta et al., 2015). Our data indicates significant and prolonged involvement of active caspases -3 and -7 in NK3.3 EV treated tumor cells.

Immunoblot analysis demonstrated that while NK3.3 EVs do not carry full-length or cleaved fragments of caspases -3, -7, or -8, they carry full-length caspase-9. By transport of caspase-9 into the target cell, NK3.3 EVs may provide a surplus of enzymatic building material for continuous construction of apoptosome structures to perpetuate caspase-dependent apoptotic activity. Additionally, we show reduced expression of Bcl-2 after NK3.3 EV-treatment of K562 cells. This is consistent with well-documented evidence that granzyme B has a role in initiating and prolonging apoptosis via direct activation of caspases and induction of mitochondrial membrane damage (Ben Safta et al., 2015).

In addition to granzyme B, granzyme A expression is also highly expressed in NK3.3 EVs. Granzyme A primarily induces mitochondrial damage in a caspase-independent manner resulting in production of reactive oxygen species (Lieberman, 2010). Studies have shown granzyme A activity in NK EVs but its role in tumor killing has not been delineated (Wu et al., 2019). Further investigation is ongoing to determine the extent to which granzyme A contributes to killing by NK3.3 EVs.

We also show that granulysin, another cytolytic effector molecule, is packaged in NK3.3 EVs. We identified the 15kDa granulysin precursor, which would need to be modified to its 9kDa active form after target cell entry for function. Granulysin has been shown to damage both the plasma and mitochondrial membranes of several tumor types by causing an influx of Ca^2+^ and an efflux of K^+^ from the cell. ER stress is also induced, resulting in caspase-7 activation followed by apoptosis without mitochondrial involvement (Okada and Morishita, 2012). We are currently investigating the potential role of granulysin in NK EV-mediated tumor death.

NK3.3 EV studies are in progress to determine the best method to obtain EVs with the strongest anti-tumor activity and the least non-specific activity. Treatments with IL-12, IL-15, or IL-21 alone or in combinations with IL-2 have been used to enhance NK cell cytotoxicity and expansion. Most recently, cytolytically enhanced exosomes, isolated from IL-15-treated NK-92MI cells, have been used to treat glioblastoma, breast cancer, and thyroid cancer in mice (Zhu et al., 2019). We are currently investigating the most effective stimulation of NK3.3 cells to produce EVs with maximum cytolytic activity.

NK3.3 EVs have a strong anti-tumor effect on every tumor cell line we tested to date, including K562, Jurkat, MDA-MB-231, MCF7 and several multiple myeloma cell lines. Many tumor cells develop mechanisms to avoid killing by evading recognition by immune cells or by becoming resistant to first-line chemotherapy drugs. Patients with triple negative breast cancer have limited treatment options as there is no molecular therapeutic target. This disease disproportionately affects young women of color (Gupta et al., 2020). Additional *in vivo* studies are ongoing to further analyze the ability of NK3.3- derived EVs to inhibit MDA-MB-231 breast tumor growth, using larger cohorts of mice, different routes of EV administration, and monitoring both primary tumor growth and metastasis. Identification of additional tumors susceptible to NK EV-mediated killing is also in progress. We propose that NK3.3 EVs may be a safe and effective new form of immunotherapy due to their ability to efficiently reduce tumor burden by selectively targeting and killing tumor cells while sparing normal cells.

## Data Availability Statement

The data from RNA sequencing of NK3.3 EVs have been deposited in the NCBI Sequence Read Archive (SRA) repository with the accession number PRJNA742047 and run number SRR14934743. The mass spectrometry proteomics data of NK3.3-derived EVs have been deposited in the ProteomeXchange Consortium via the PRIDE partner repository with the dataset identifier PXD027051 and DOI: 10.6019/PXD027051. The proteomics data for control and EV-treated K562 cells can be accessed by identifier PXD027221 and DOI: 10.6019/PXD027221.

## Ethics Statement

The animal study was reviewed and approved by Saint Louis University and St. Louis VA Animal Care and Use Committees.

## Author Contributions

AC performed the experiments and data analysis and wrote the manuscript. JK designed the project, reviewed the data and manuscript and supervised the study. Both authors contributed to the article and approved the submitted version.

## Conflict of Interest

The authors declare that the research was conducted in the absence of any commercial or financial relationships that could be construed as a potential conflict of interest.

## Publisher’s Note

All claims expressed in this article are solely those of the authors and do not necessarily represent those of their affiliated organizations, or those of the publisher, the editors and the reviewers. Any product that may be evaluated in this article, or claim that may be made by its manufacturer, is not guaranteed or endorsed by the publisher.
