# Supplementary material for: Extracellular Vesicles From the Human Natural Killer Cell Line NK3.3 Have Broad and Potent Anti-Tumor Activity
Source: Front Cell Dev Biol. 2021 Jul 23;9:698639. doi: 10.3389/fcell.2021.698639 (PMC8343581; doi:10.3389/fcell.2021.698639)
Supplement: Supplementary file 1 [file Table_1.DOCX]

**SUPPLEMENTAL TABLES AND FIGURES**

**Table S1.** Proteomic analysis of NK3.3-derived EVs

**Table S2.** RNA profiling of NK3.3-derived EVs

**Table S3.** Proteomic analysis of untreated, NK3.3- and HEK293 EV-treated K562 cells

**Figure S1.** **NK EV** **particle size characterization.** NTA was used to evaluate vesicle size distribution of different sample preparations. (A) NK-92 derived EVs were isolated using ExoQuick precipitation. (B) NK3.3-derived were isolated using ExoQuick precipitation. (C) NK3.3-derived exosomes were isolated by differential centrifugation at 2,000 x g, 10,000 x g, followed by 100,000 x g. (D) NK3.3-derived microvesicles (MVs) isolated by 10,000 x g ultracentrifugation. Data are representative of 3 measurements.

**Figure S2. Characterization of NK3.3-derived EVs.** (A) NK and HEK293 EV lysates were compared with whole cell lysates using a negative control protein (cytochrome c) and a positive control protein (Tsg101) to assess purity of the EV preparations. (B) Lysates of HEK293-, NK3.3-and NK-92 EVs, and HEK293- and NK3.3-whole cell lysates were analyzed for protein expression. Each lane contains 25μg of protein. A molecular weight standard separates EV lysates from cell lysates (lane 4). (C) Gene ontology annotation generated from EV proteomic analysis identified the frequency of cellular components associated with NK3.3 EV proteins. (D) The frequency of the RNA species in NK3.3 EVs was determined by qPCR quantitation and high output single-end sequencing.

**Figure S3. Changes in K562 morphology due to NK3.3 EV treatment.** K562 cells treated with either PBS, 2.5μM staurosporine, or 100μg/ml of NK3.3 EVs were observed and imaged using phase microscopy at 200x magnification. One representative experiment of 7 is shown. Scale bar: 50μm.

**Figure S4. Decreased metabolic activity, proliferation, and induction of apoptosis in NK3.3 EV-treated K562.**  Cells were treated with 100μg/ml NK3.3 EVs isolated by either ExoQuick precipitation or differential ultracentrifugation. (A) Cell viability was assessed using the WST-1 cell viability assay. (B) Corresponding live cell counts were determined by automated cell counting using trypan blue dye exclusion. Mean cell numbers ± SE; n=3.  *p-*values determined by comparison of NK3.3 EV-treated cells to PBS-treated cells. ***** = p ≤ 0.05, ****** = p ≤ 0.005, ******* = p ≤ 0.0005. (C) K562 and healthy cord blood lymphocytes (CB) were treated with either PBS or NK3.3 EVs (20μg/ml) and a luminescent substrate using the RealTime-Glo MT cell viability assay. Cells were continuously monitored over 89 hours. RLU: relative luminescence unit. A total of 6 assays were performed; one representative experiment is shown. (D) K562 cells treated with either PBS, 100μg/ml HEK293 EVs, 100μg/ml NK3.3 EVs, or 2.5μM staurosporine for 12, 24, 48, and 72 hours were incubated with a DEVD pro-luciferin substrate that produces a luminescent signal upon cleavage by caspases -3 and -7. Mean RLU ± SE; n=6.  *p-*values determined by comparison of NK3.3 EV-treated cells to PBS- and HEK293 EV-treated cells. *** = p ≤ 0.001
