# Supplementary material for: Extracellular Vesicles From the Human Natural Killer Cell Line NK3.3 Have Broad and Potent Anti-Tumor Activity
Source: Front Cell Dev Biol. 2021 Jul 23;9:698639. doi: 10.3389/fcell.2021.698639 (PMC8343581; doi:10.3389/fcell.2021.698639)

Figure 2
30ug/lane

Lane 1: 293
Lane 2: 3.3
Lane 3: NK92

Common EV Proteins

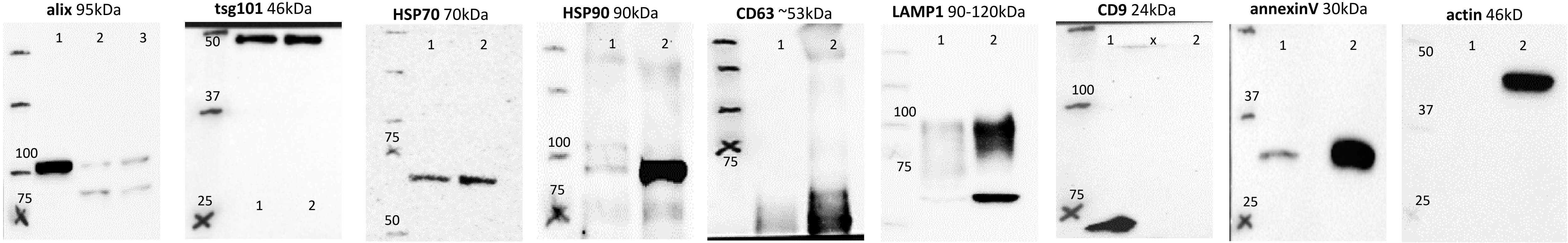

NK-Specific Proteins

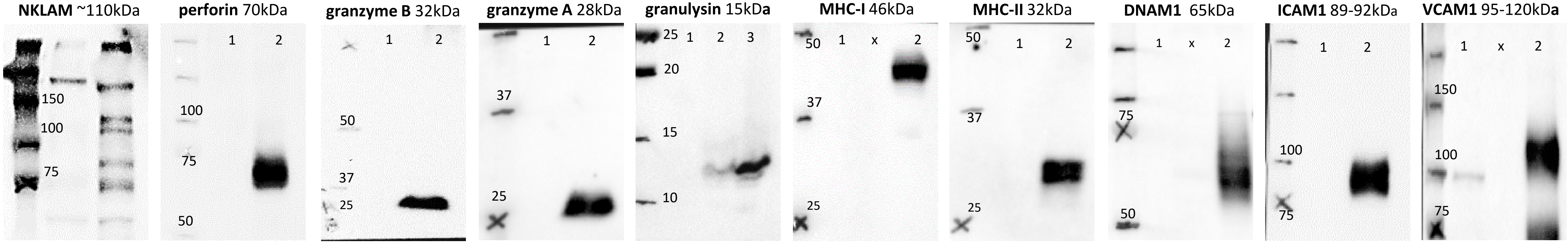

Figure 3

20ug/lane

Lane 1: 0
Lane 2: +p
Lane 3: +p+s

Cytoplasmic

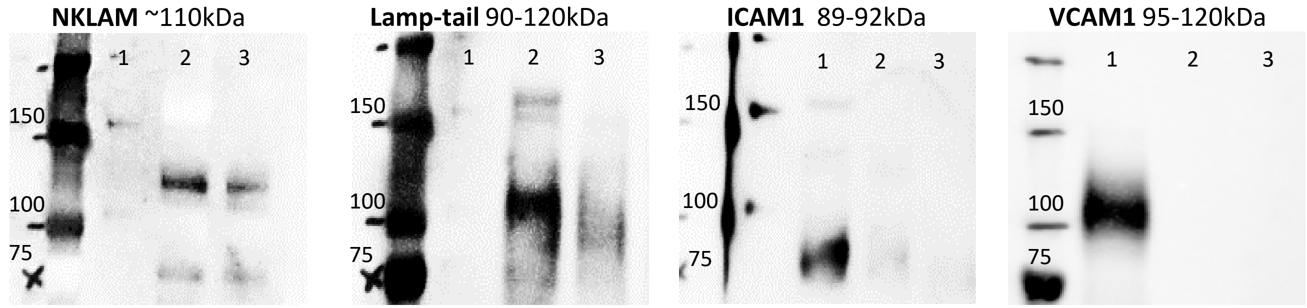

Lumenal

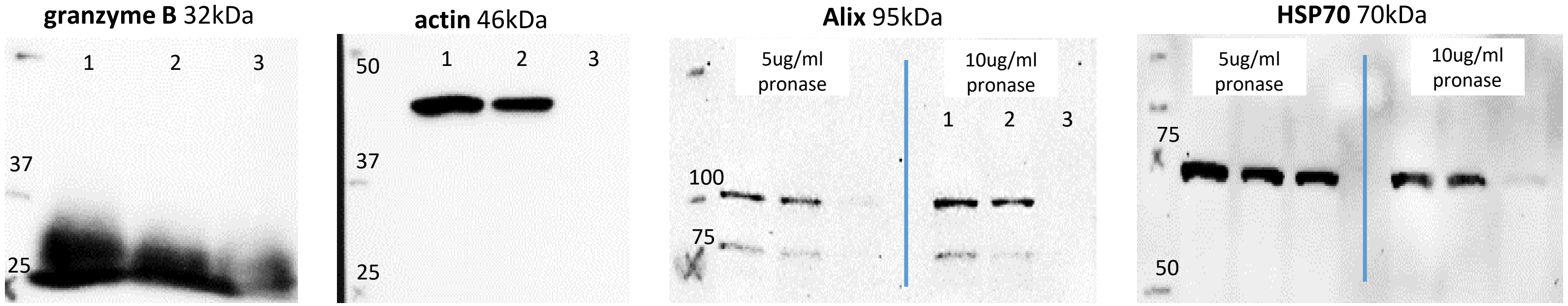

Figure 6 (E)

25ug/lane

			NK EV	
	PBS	St	100	25
Lanes:	1	2	3	4

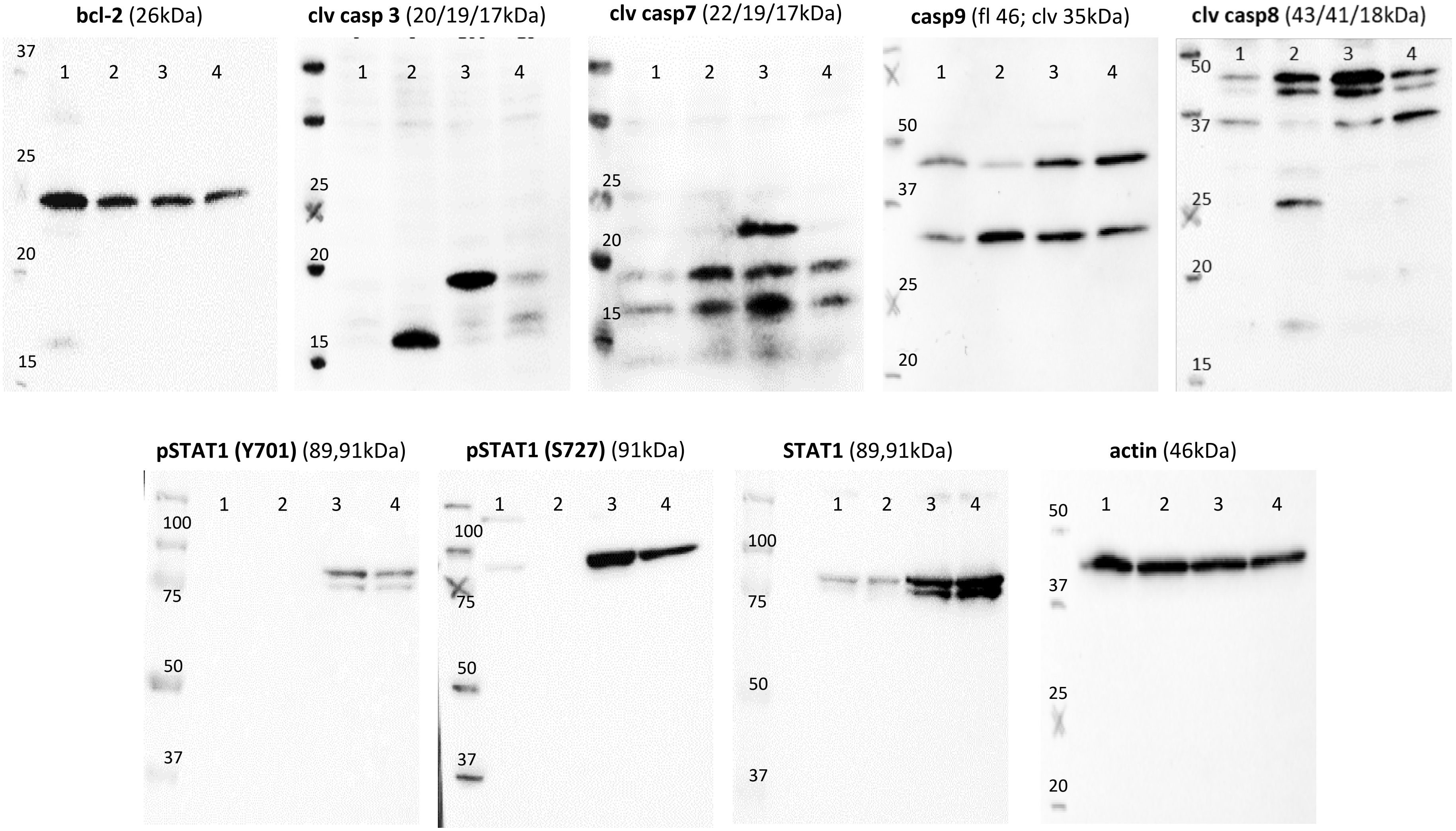

Figure 6 (F)

20ug/lane

			K562lys
	3.3EV	NK3.3lys	(treated)
Lanes:	1	2	3

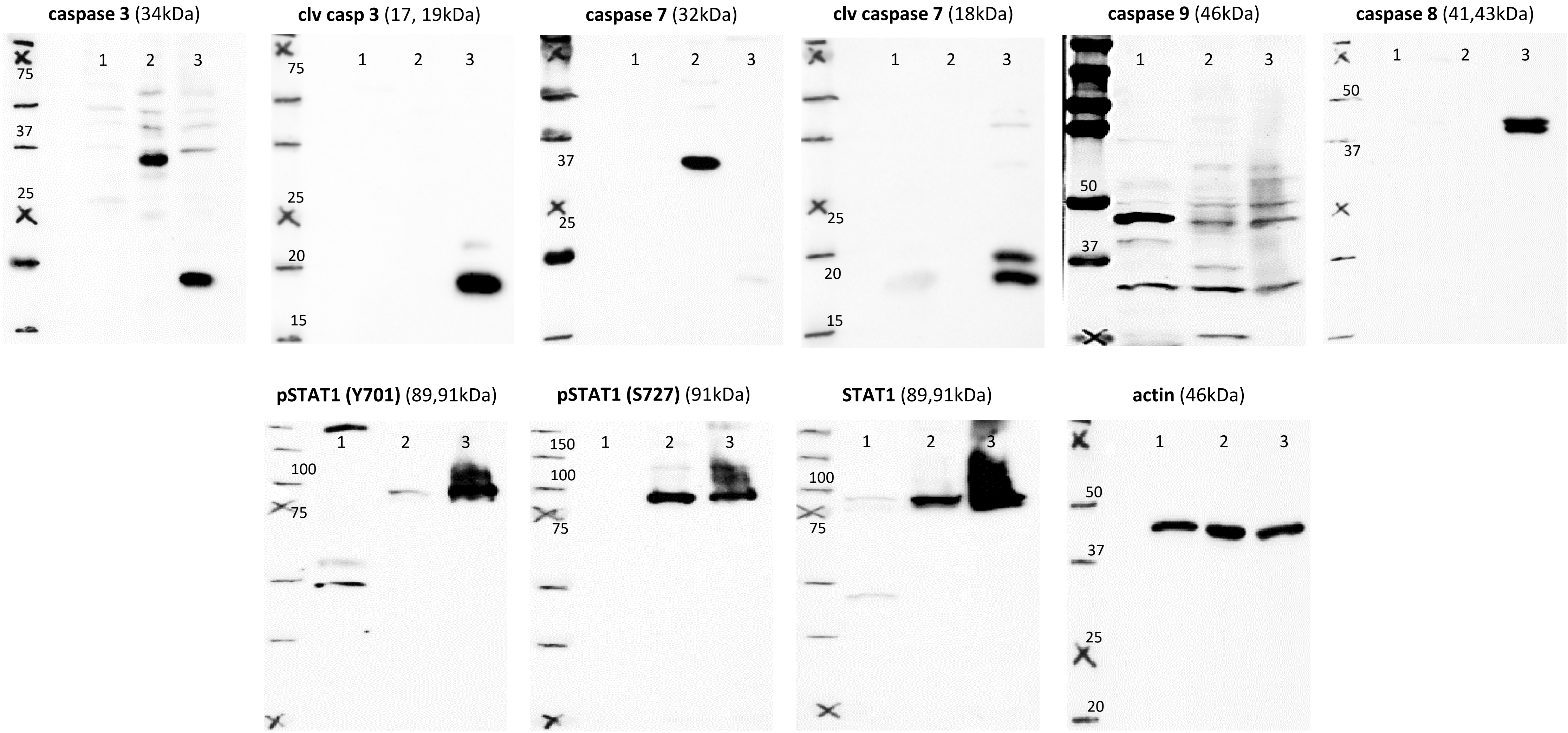

Supplemental Figure 2

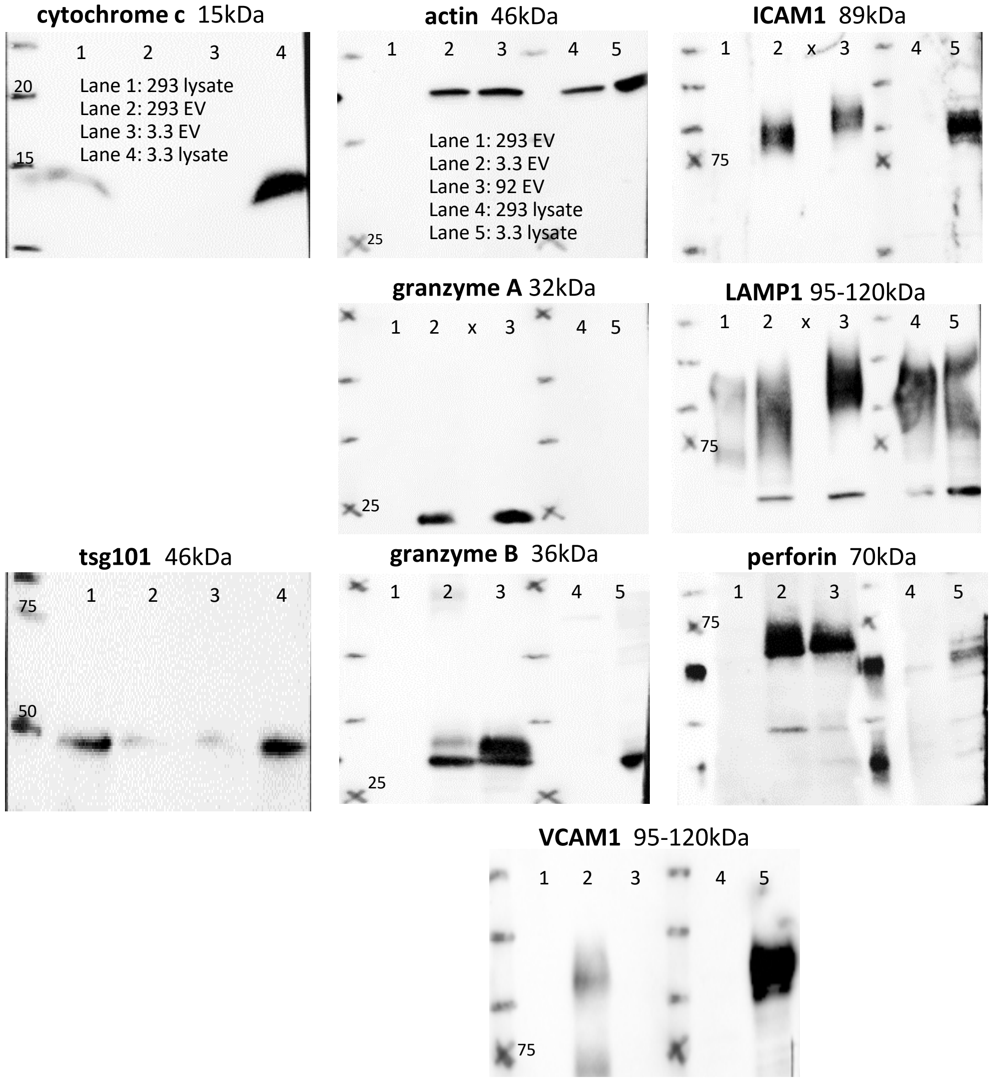

Supplement: Supplementary file 5 [file Image_1.pdf]
